# Alterations to the Lung Microbiome in Idiopathic Pulmonary Fibrosis Patients

**DOI:** 10.3389/fcimb.2019.00149

**Published:** 2019-05-21

**Authors:** Xunliang Tong, Fei Su, Xiaomao Xu, Hongtao Xu, Ting Yang, Qixia Xu, Huaping Dai, Kewu Huang, Lihui Zou, Wenna Zhang, Surui Pei, Fei Xiao, Yanming Li, Chen Wang

**Affiliations:** ^1^Department of Respiratory and Critical Care Medicine, National Center of Gerontology, Beijing Hospital, Beijing, China; ^2^Clinical Biobank, National Center of Gerontology, Beijing Hospital, Beijing, China; ^3^Department of Laboratory Medicine, Beijing Hospital, Beijing, China; ^4^National Clinical Research Center for Respiratory Diseases, Center for Respiratory Diseases, China-Japan Friendship Hospital, Peking University Health Science Center, Beijing, China; ^5^Department of Pulmonary and Critical Care Medicine, China-Japan Friendship Hospital, Peking University Health Science Center, Beijing, China; ^6^Department of Respiratory and Critical Care Medicine, Bengbu University Affiliated Hospital, Bengbu, China; ^7^Department of Respiratory and Critical Care Medicine, Beijing Chao-Yang Hospital, Capital Medical University and Beijing Institute of Respiratory Medicine, Beijing, China; ^8^The Key Laboratory of Geriatrics, National Center of Gerontology, Beijing Hospital, Beijing, China; ^9^Annoroad Gene Technology (Beijing) Co., Ltd., Beijing, China; ^10^National Clinical Research Center for Respiratory Diseases, Peking Union Medical College, Chinese Academy of Medical Sciences, Beijing, China

**Keywords:** idiopathic pulmonary fibrosis, bronchoalveolar lavage fluid, microbiota, antibiotic resistant gene, virulence factor

## Abstract

Lung microbiome ecosystem homeostasis in idiopathic pulmonary fibrosis (IPF) remains uncharacterized. The aims of this study were to identify unique microbial signatures of the lung microbiome and analyze microbial gene function in IPF patients. DNA isolated from BALF samples was obtained for high-throughput gene sequencing. Microbial metagenomic data were used for principal component analysis (PCA) and analyzed at different taxonomic levels. Shotgun metagenomic data were annotated using the KEGG database and were analyzed for functional and metabolic pathways. In this study, 17 IPF patients and 38 healthy subjects (smokers and non-smokers) were recruited. For the PCA, the first and the second principal component explained 16.3 and 13.4% of the overall variability, respectively. The β diversity of microbiome was reduced in the IPF group. Signature of IPF's microbes was enriched of *Streptococcus, Pseudobutyrivibrio, and Anaerorhabdus*. The translocation of lung microbiome was shown that 32.84% of them were from oral. After analysis of gene function, ABC transporter systems, biofilm formation, and two-component regulatory system were enriched in IPF patients' microbiome. Here we shown the microbiology characteristics in IPF patients. The microbiome may participate in altering internal conditions and involving in generating antibiotic resistance in IPF patients.

## Introduction

Idiopathic pulmonary fibrosis (IPF) is a progressive, fatal disease without a known cause (Molyneaux et al., 2014). IPF is increasing in prevalence and has a median survival of 3 years after diagnosis; the life expectancy for IPF patients is worse than those of some cancers (Maher et al., 2007; Ley et al., 2011; Navaratnam et al., 2011; Molyneaux et al., 2014). Because microorganisms may disturb the internal environment of the lower airway and cause lung damage, it is crucial to determine the precise composition of the lung microbiota and predict associated gene functions to understand IPF (Han et al., 2014; O'dwyer et al., 2016).

Traditional microbiological culture-based methods do not provide a complete profile of the microbiota in the lower respiratory tract (Garg et al., 2017; Ubags and Marsland, 2017). In contrast, culture-independent approaches, primarily based on gene sequencing, better describe the wide diversity of the microbial inhabitants of the lung microbiota and are able to identify significant differences between healthy subjects and patients with various respiratory diseases (Turnbaugh et al., 2007; Huse et al., 2012; Dickson and Huffnagle, 2015; O'dwyer et al., 2016).

Bronchoalveolar lavage fluid (BALF) samples obtained from the lower respiratory tract are used for routine diagnostic procedures. BALF sampling is suitable for the identification of a distinct microbiome in the lower respiratory tract (Zemanick et al., 2017). The bronchi harbor a specific microbiome associated with healthy individuals as well as a microbiome specific to pulmonary fibrosis patients (Charlson et al., 2011; Zimmermann et al., 2015). In addition, some studies have identified differences in the microbiomes of the upper respiratory tracts of smokers vs. non-smokers (Morris et al., 2013; Faner et al., 2017). Smoking may directly affect the composition of the respiratory microbiome, leading to changes or shifts in microbial community structure (Charlson et al., 2010; Sapkota et al., 2010; Lee et al., 2012). However, few studies have examined differences in the microbiomes of the lower respiratory tract in smokers vs. non-smokers (Garmendia et al., 2012; Morris et al., 2013).

In our current study, we broadly characterized the BALF-associated microbial communities in IPF patients and healthy individuals, including smokers and non-smokers. Additionally, we compared the lung microbiome with other local microbial communities, including those of the upper airway tract (the oropharynx) and gut. We attempted to assess the usefulness of shotgun metagenomics applied directly to DNA extracted from BALF samples to characterize the lung microbiomes in IPF and healthy patients and to reveal the role of the microbiome in IPF pathophysiology.

## Methods

### Study Design

A diagnosis of IPF was made after multidisciplinary consultations. Only individuals diagnosed according to the international guidelines of the American Thoracic Society (ATS) and the European Respiratory Society (ERS) were subsequently included in this study (Travis et al., 2013). Healthy control subjects included non-smokers and smokers with normal lung function. Exclusion criteria included: (a) a history of self-reported upper or lower respiratory tract infection in the previous 3 months; (b) antibiotic use in the previous 3 months; (c) acute IPF exacerbation; and (d) other respiratory disorders. Written informed consent was obtained from all subjects, and the study was approved by the Institutional Review Board and Ethics Committee of Beijing Hospital (Beijing, China). The workflow of this research is shown in Supplementary Figure 1.

### Bronchoscopy

**Figure 1:**
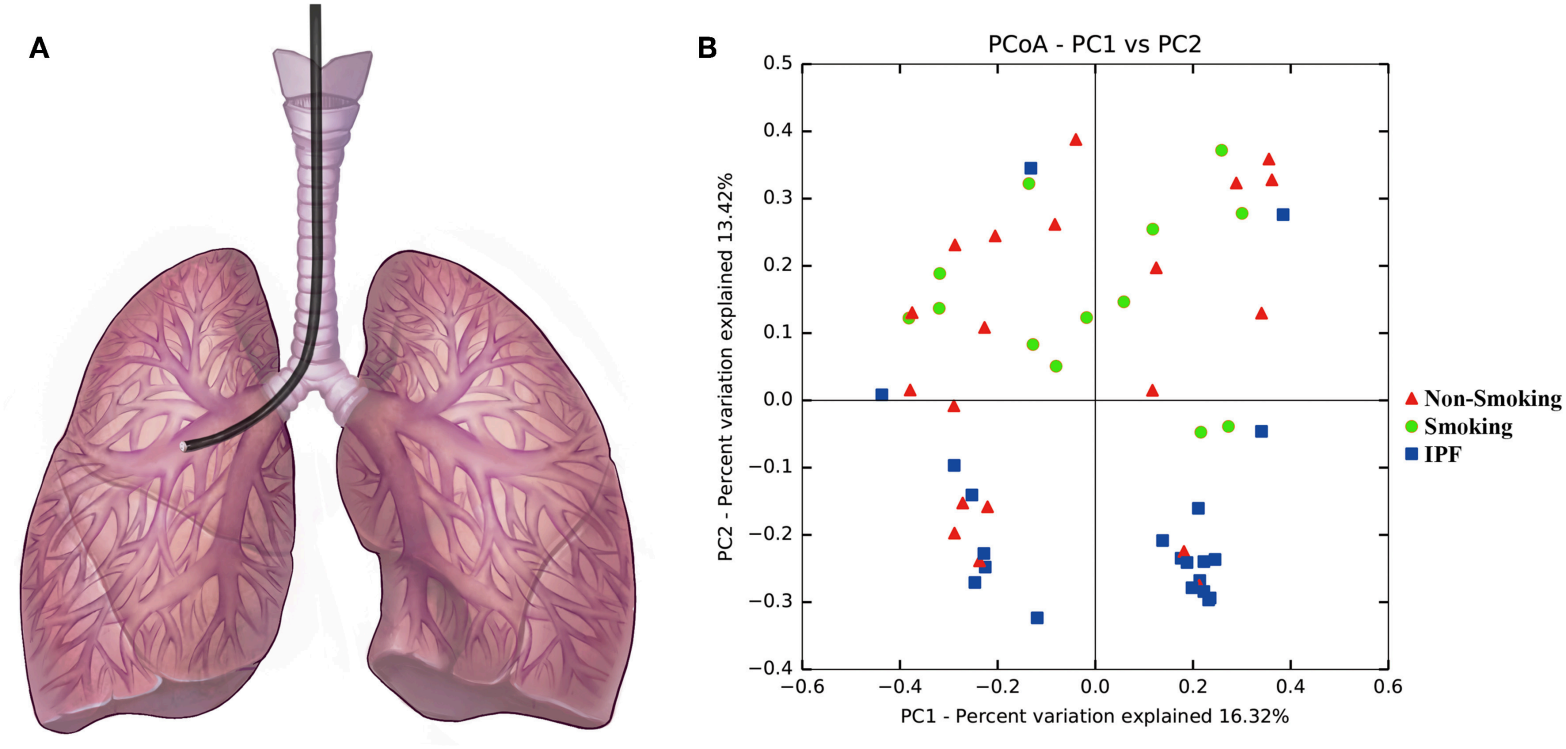
Principal component analysis with BALF samples from IPF patients and health subjects. **(A)** Diagram of the bronchoscopy performance. **(B)** Principal component analysis with BALF samples from IPF patients and health subjects (including smokers and non-smokers) was performed based on the taxonomic profiles at species level. Different colors of points were represented for different groups: the blue points represent BALF samples from IPF group, the green points represent BALF samples from smoking control subjects, and the red points represent BALF samples from non-smoking control subjects. BALF samples from IPF patients were with an apparent clustering pattern of microbial composition compared with smoking control subjects, but no significant distribution pattern with the non-smoking control subjects.

Fiberoptic bronchoscopies with bronchoalveolar lavage (BAL) were performed according to ATS guidelines via the oropharyngeal route in accordance with a standard operating procedure, during which 20 ml of normal saline was instilled into a designated segment of the lobe showed in Figure 1A (Busse et al., 2005). After specimen collection, an aliquot of unfiltered and unprocessed BAL was immediately placed on ice and then frozen at −80°C for further analysis.

### DNA Extraction

DNA was extracted from 3.0 ml of each original BALF sample using a QIAamp DNA Microbiome Kit (catalog 51704, Qiagen, Hilden, Germany) in strict accordance with the manufacturer's instructions. The DNA concentration was measured using a Qubit® 2.0 Fluorimeter (Life Technologies, Invitrogen, USA).

### DNA Library Construction and Sequencing

DNA libraries were constructed using an Illumina TruSeq DNA kit according to the manufacturer's instructions. The same Illumina workflows were used to perform cluster generation, template hybridization, isothermal amplification, linearization, blocking, denaturation and hybridization of the sequencing primers. We performed paired-end sequencing on 2 × 100 base pairs (bp) or single-end sequencing on 100 bp. The base-calling pipeline was used to process raw fluorescent images and to call sequences. Reads that mapped to the human genome (hg19) were removed from each sample with BWA MEM using the default parameter (Li and Durbin, 2009). For quality control, we employed Trimmomatic (version 0.36) with the following criteria: (a) scan the read with a 4-base-wide sliding window, cutting when the average quality per base drops below 15; (b) drop reads below 50 bases in length; and (c) remove leading/tailing low quality (*Q* < 13). This produced an average of 8.5 gigabases (Gb) of high-quality sequences for each sample, resulting in a total of 470 Gb of sequence data (Supplementary Table 1). The reads were assembled into contigs for all samples using the assembly software IDBA (version 1.1.1) (Peng et al., 2012). IDBA was applied for Illumina short read assembly with the parameters “–pre_correction –mink 30 –maxk 120 –step 10.” Then, we removed ambiguous bases from the assembled scaffolds and discarded scaffolds with lengths less than 500 bp. Finally, 73% of the total reads were used to generate 88,761 contigs without ambiguous bases (minimum length of 500 bp). These contigs had a total length of 210,670,314 bp, an average N50 length of 4,151 bp and ranged from 503 to 620,529 bp.

### Construction of a Non-redundant Microbiome Gene Set

To predict microbial genes from the assembly, we applied the methodology used in the MetaHIT human gene catalog study (Qin et al., 2010). MetaGeneMark (MetaGeneMark_v1.mod) was used to predict the open reading frames (ORFs) in contigs without ambiguous bases (Zhu et al., 2010). The program predicted 250,268 ORFs using a 100-bp cut-off. The total length of the predicted ORFs was 183,986,409 bp, representing 87.3% of the total contig length. Among these ORFs, 142,871 (57.1%) were complete genes, while 107,397 (42.9%) were incomplete without a start or stop codon. A non-redundant “gene set” was established by removing redundant ORFs, defined as those sharing 95% identity with VSEARCH (Rognes et al., 2016). Then, genes from the HMP and HOMD databases (Dewhirst et al., 2010) were merged as a non-redundant reference gene set for gene-source analysis.

### Organism Abundance Profiling

We followed the approach described in previous study to perform organism abundance profiling (Qin et al., 2014). BWA was applied to align paired-end clean reads against reference genomes using default parameters. Reads with alignments on the same reference genomes were assigned into the following two groups: reads having alignments with only one genome were identified as unique reads (U), and reads having alignments with more than one genome were identified as multiple reads (M).

For species S, if its abundance was Ab (S), and if it potentially aligned with U unique reads and M multiple reads, the following formulae were used:

$$\begin{array}{l} \text{Ab}\left(\text{S}\right)=Ab\left(U\right)+Ab\left(M\right)\\ \text{Ab}\left(\text{U}\right)=U/l\\ \text{Ab}\left(\text{M}\right)=\left(\overset{M}{\underset{i=l}{\sum }}Co*\left\{M\right\}\right)/l \end{array}$$

Shannon diversity index (H) at the species level was calculated as the following, with *p*_*i*_ denoting the proportion in group *k*.

$$\begin{array}{l} H=-\overset{k}{\underset{i=1}{\sum }}p_{i}\log\left(p_{i}\right) \end{array}$$

### Gene Abundance Profiling

Gene abundance was determined using a method similar to RPKM (reads per kilobases per million reads) used to quantify gene expression based on RNA sequencing data. In brief, high-quality original Illumina reads from each sample were aligned with a reference gene set using BWA. For each gene, G_i_, the number of read pairs that aligned to it divided by the length of the gene was calculated as Num_G_i_, and the relative abundance, RNum_G_i_, of each gene in each sample (n genes) was computed using the following formula:

$$\begin{array}{l} \text{RNu}{\text{m}}_{\text{Gi}}=Num\text{\_}Gi/\overset{n}{\underset{i=1}{\sum }}Num\text{\_}Gi \end{array}$$

### Gene Function Classification and Ortholog Group Abundance Profiling

Protein sequences of the predicted genes were searched using BLASTp (Altschul et al., 1990) against the EggNOG 4.5 database (Huerta-Cepas et al., 2016) (Supplementary Table 3) and the KEGG gene database(Kanehisa et al., 2017) with the parameters “-num_descriptions 10,000, -*e*-value 1e-5.” Genes that had alignments with a score slightly higher than 60 were assigned into one or more eggnog or KEGG ortholog groups. We used the methods introduced in previous study (Li et al., 2014) to calculate the abundances of KEGG ortholog groups; additionally, we added the abundances of proteins assigned into the same KEGG ortholog groups as abundances of KEGG ortholog groups, and KEGG ortholog group profiles were generated.

### Quantification of Antibiotic Resistance Genes and Virulence Factors

Antibiotic resistance genes (ARGs) were annotated using the ARDB (Liu and Pop, 2009) database with BLASTp (*e*-value < 1 × 10^−5^). Then, antibiotic resistance gene abundance was determined as mentioned above. We used the Wilcoxon rank sum test to determine significantly different abundances between conditions. Virulence factors (VFs) were analyzed using a similar strategy. In brief, all VFs were identified using the VFDB database (Chen et al., 2016) with BLASTp (*e*-value < 1 × 10^−5^). Gene abundances and related statistical analysis were followed the ARGs' method.

### Multivariate Analysis

To analyze the beta diversity of various groups based on the Bray-Curtis dissimilarity, ANOSIM was performed to detect significant dissimilarity or similarity in community composition among different groups (IPF/smoking/non-smoking). Pairwise comparisons were performed in ANOSIM, and pairwise *P*-values were obtained to assess significant differences in community composition between any two groups.

### Statistical Analysis

PCA was analyzed using QIIME (Version 1.9.1) (Caporaso et al., 2010). Differential gene abundance and KEGG modules were tested with the Wilcoxon rank-sum test. One-way ANOVA test was performed and *P*-values were corrected for multiple testing with the Benjamin & Hochberg method using R software Version 3.1.1 (http://www.r-project.org/).

### Availability of Data

The data set supporting the results of this article has been deposited in the NCBI Short Read Archive database under BioProject accession code PRJNA387212.

## Results

### Patients Characteristics

In total, 17 IPF patients (IPF group) and 38 healthy control subjects (control group, including 23 non-smokers and 15 smokers) were included according to the inclusion criteria. The demographic data for these subjects are summarized in Table 1. IPF groups were slightly older than healthy subjects (IPF group vs. control group: [62.71 ± 7.90] years vs. [61.28 ± 9.37] years). As expected, IPF patients exhibited reduced forced expiratory volume in 1 one second (FEV1) and forced vital capacity (FVC) compared with those of healthy subjects. Total lung volume (TLC), residual lung volume (VR), lung diffusing capacity (DLCO) and the transfer coefficient (KCO) were sharply reduced in the IPF group, shown in Table 1.

**Table 1:** Patient characteristics.

	**Healthy control (*n* = 38)**	**IPF patients (*n* = 17)**
Age, years	62.71 ± 7.90	61.28 ± 9.37
Gender (M/F)	24/14	11/6
Smoker (Never/current), n	23/15	11/6
Pack-year	14 ± 6	48 ± 32
FEV1% pred	110 ± 10	68 ± 17
FVC% pred	115 ± 14	66 ± 16
FEV1/FVC%	82 ± 5	77 ± 12
TLC, %pred	nd	65 ± 13
RV, %pred	nd	65 ± 30
DLCO% pred	nd	34 ± 12
KCO% pred	nd	54 ± 14

### Principal Component Analysis for Microbiome

To identify any differences in the organismal structure of the lung microbiota, principal component anlaysis was performed based on taxonomic profiles at the species level (Figure 1B). Different colored points represent different groups: the blue points represent BALF samples from the IPF group, the green points represent BALF samples from smoking control subjects, and the red points represent BALF samples from non-smoking control subjects. The first principal component explained 16.32% of the overall variability among different groups, whereas the second principal component explained 13.42% of variability. As shown in Figure 1B, an apparent microbial composition clustering pattern was identified for IPF patients and smoking control subjects. Conversely, non-smoking control subjects, which are marked in red, exhibited no significant distribution pattern.

### Microbial Community Structure of BALF

**Figure 2:**
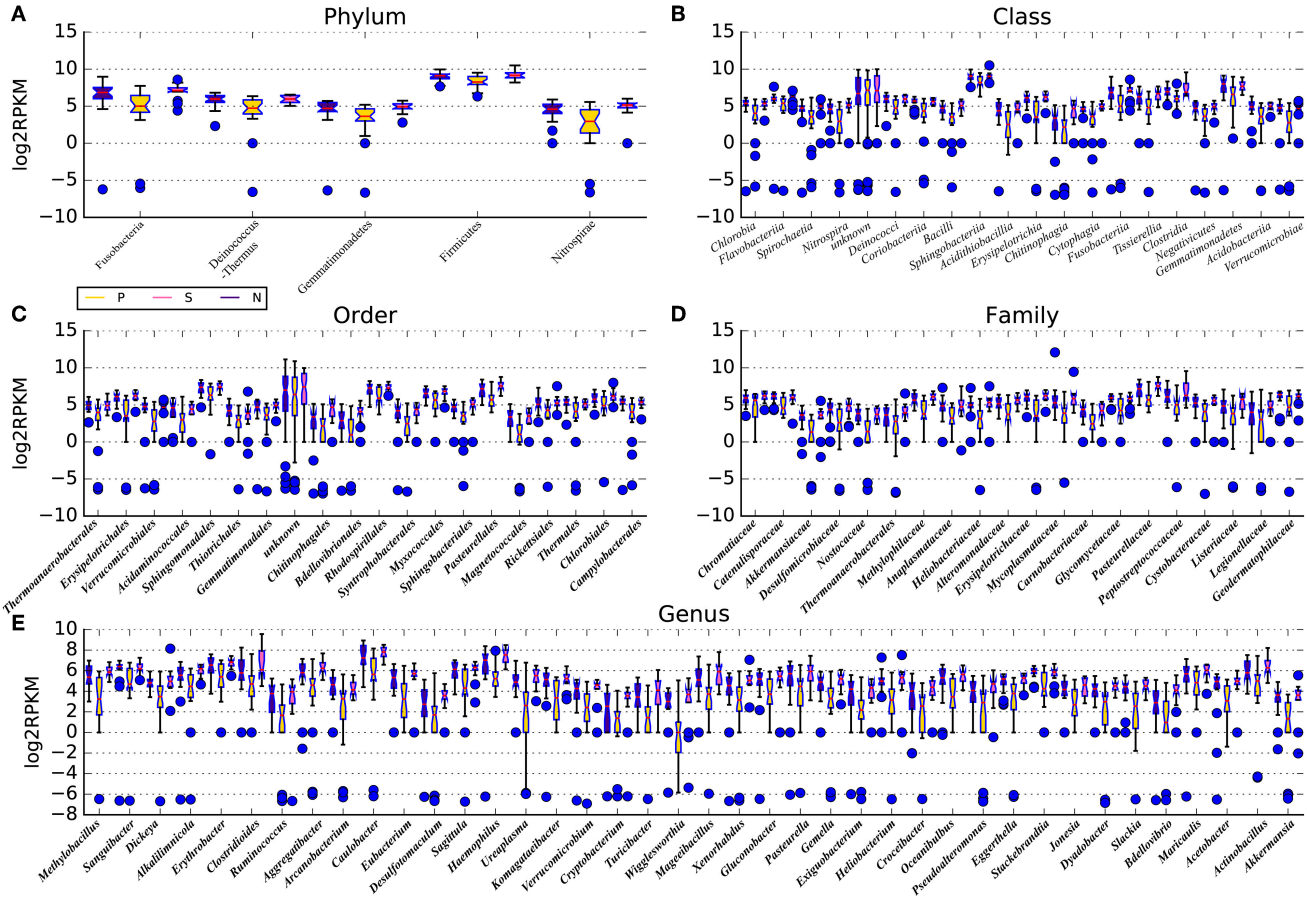
Microbiota composition of BALF samples in IPF and healthy subjects. The microbiota in IPF patients and healthy subjects (including smokers and non-smokers) were shown at the phylum **(A)**, class **(B)**, order **(C)**, family **(D)**, and genus **(E)** levels. Species with a median relative abundance larger than 0.01% of the total abundance in either the control group or the IPF group were included for comparison. P stands for IPF group, N stands for non-smoking normal subjects, S stands for smoking normal subjects. All results are presented as the median, the 25–75 % percentiles and the variation range; results for IPF group (P) are presented as yellow boxes, smoking control subjects (S) are marked with pink boxes and non-smoking control subjects (N) are marked with violet boxes. Blue dots represent the abnormal observations at the corresponding taxonomic levels.

Sequencing reads (average number: 7,242,099 ) were aligned against 3,096 reference genomes from the National Center for Biotechnology Information and the HMP reference sequence, which contains 131 archaeal strains comprising 97 species, 326 lower eukaryotes comprising 326 species, 3,683 viral strains comprising 1,420 species, and 1,751 bacterial strains comprising 1,253 species (Supplementary Table 1). Relative abundances at the phylum, class, order, family, genus, and species levels were compared between the IPF group and control group. Intrinsic differences in lung microbiota composition are shown in Figure 2 and Supplementary Table 2. Species with a median relative abundance larger than 0.01% of the total abundance in either the control group or the IPF group were included for comparison. Furthermore, we imported taxonomic data into QIIME for comparison. At the phylum level, *Firmicutes* (ANOVA, *P*-value = 0.02) and *Fusobacterium* (ANOVA, *P*-value = 0.04) dominated the BALF microbial communities in both groups (Figure 2). Compared to the control group, the IPF group had lower levels of *Bacteroidetes* (ANOVA, *P*-value = 0.03) but higher levels of *Proteobacteria* (ANOVA, *P*-value = 0.004) and *Fusobacteria* (ANOVA, *P*-value = 0.022). At the genus level (Figure 2E), *Bacteroides* was the dominant phylotype in both groups but was significantly decreased in the IPF group. Similar to previous studies (Morris et al., 2014), *Streptococcus*, representing 23.0 % of total reads, was the most common genus in subjects with IPF, followed by *Pseudobutyrivibrio, Anaerorhabdus, Campylobacter*, and *Blautia*, all of which were enriched in the IPF group. In contrast, *Sutterella, Coprococcus, Parasutterella, Paludibacter* and *Dorea* were dominant in the healthy group. The microbial communities of subjects with IPF were by contrast less diverse (Shannon diversity index, 2.81 ± 0.08 vs. 4.01 ± 0.10; *P* = 0.004) than the healthy subjects.

### Biodiversity Analysis of BALF Samples

**Figure 3:**
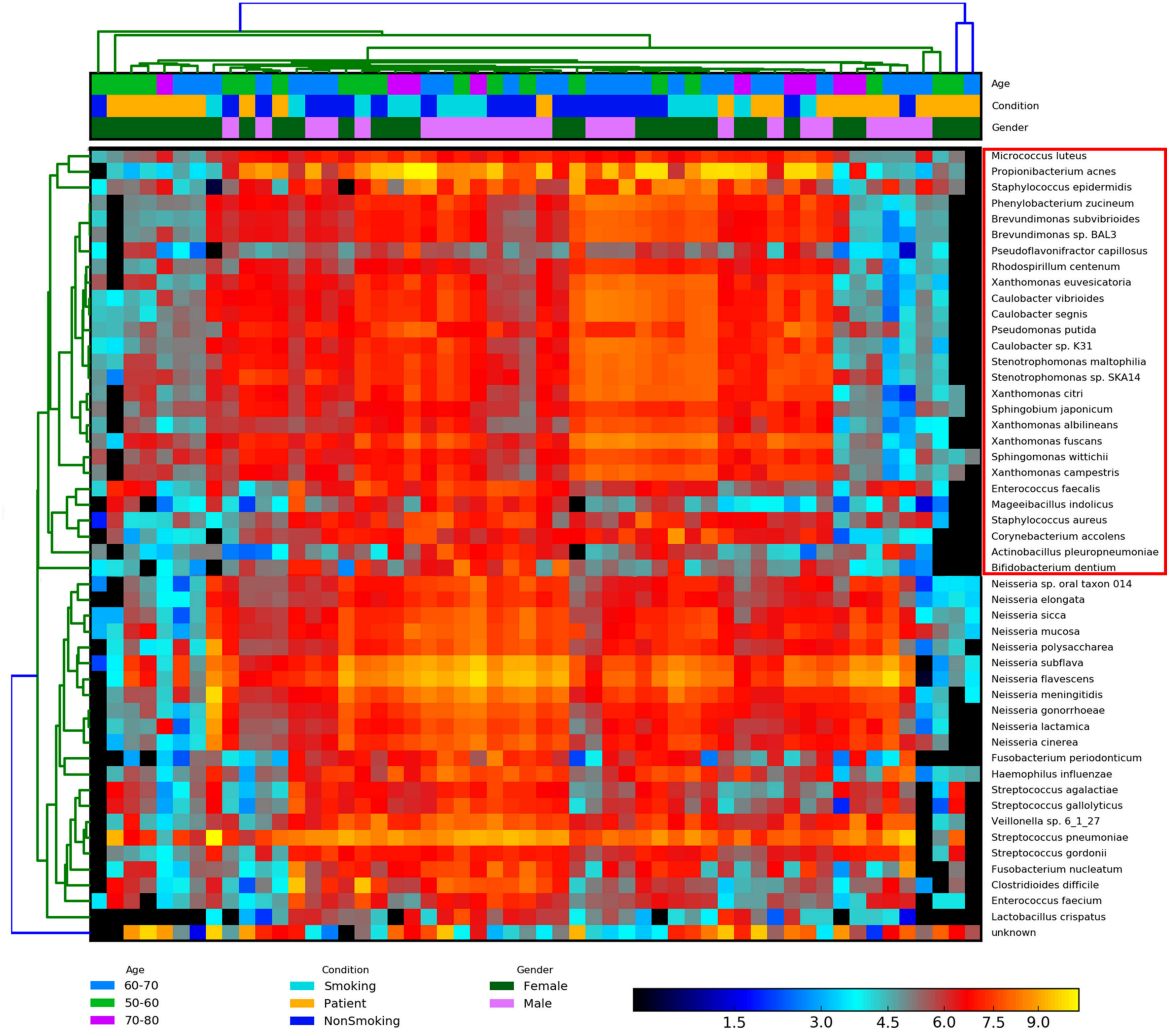
Heatmap of the normalized abundances of all BALF samples. A similar grouping pattern on the combinations of the gender, age, and condition was also identified by Bray-Curtis matrix using the hclust2 and Mantel tests. The significant correlated of community structure of two groups was revealed (*P*-value = 0.01). The species in the red box are group 1, opportunistic pathogenic bacteria primarily derived from the skin and mouth, as mentioned above. The rest species are group 2, which is mainly from gut. Heatmap is color-coded based on the normalized abundance of species, from black (lower abundance) to red (higher abundance).

To measure the diversity of the BALF communities in IPF patients and healthy subjects (including smokers and non-smokers), we employed various methods, such as Adonis and ANOSIM of QIIME, to evaluate differences (beta-diversity) in the lung microbiota among the groups. BALF biodiversity was significantly reduced (*P*-value = 0.01) in the IPF group compared with that in the smoking/non-smoking groups according to the ADONIS results. A similar grouping pattern was identified based on the Bray-Curtis matrix using hclust2 (Figure 3). We identified the following two groups that were differentiated based on combinations of sex, age, and condition: Group 1, opportunistic pathogenic bacteria primarily derived from the skin and mouth, according to study of Blauwkamp (Blauwkamp et al., 2019); and Group 2, bacteria derived from the gut. These two groups were similar to those identified by ANOSIM. Mantel tests of the vegan package revealed the significant correlation of community structure with a *P*-value = 0.001 (Mantel Statistic R: 0.3057).

### Crosstalk of BALF Microbiom to the Oral and Gut Microbiomes

**Figure 4:**
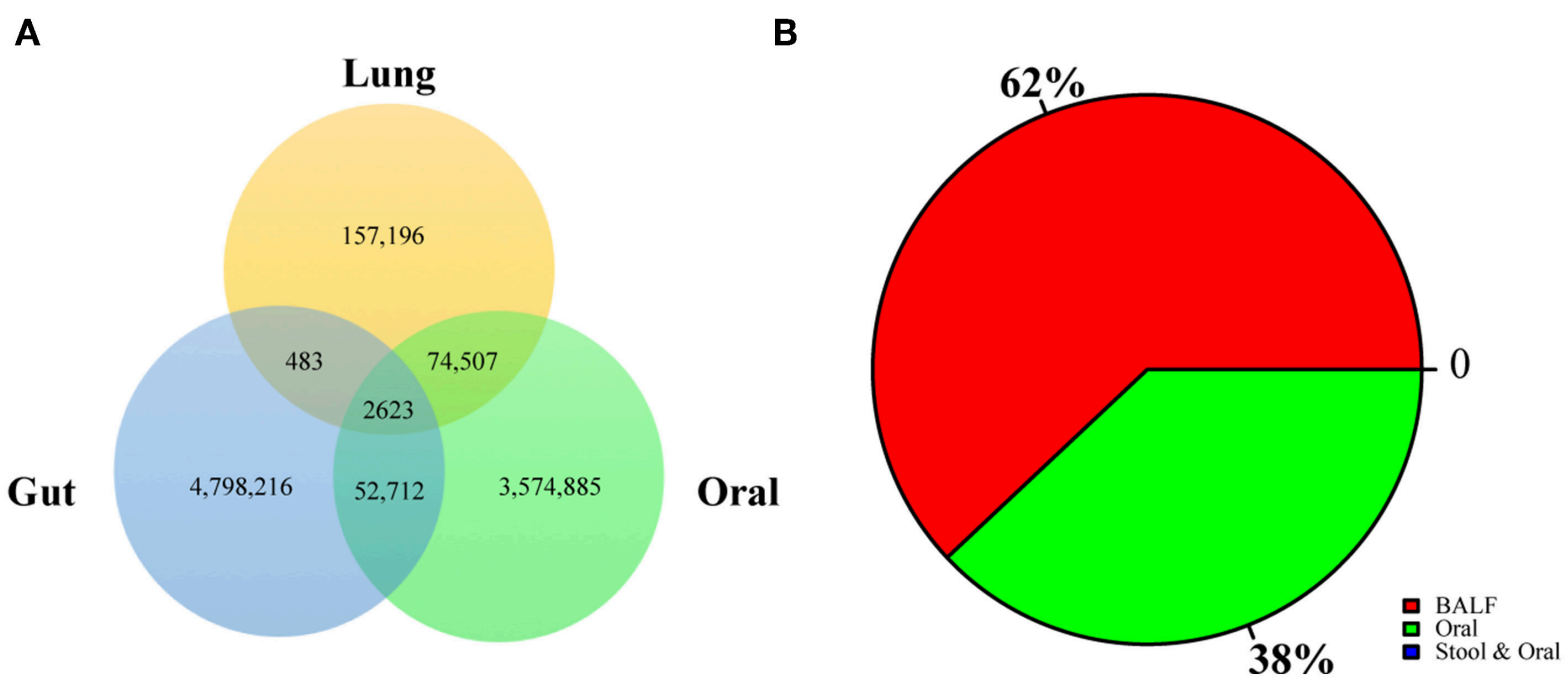
Crosstalk of BALF's microbiome to oral and gut microbiome. **(A)** The origin lung microbiome was explored by comparison with HOMD and HMP databases. The genes rescore was shown: 32.84% genes were from oral and 1.32% genes were from the gut, as determined by the Vsearch. **(B)** Crosstalk of the different enriched genes were analyzed in this research: 38% of them were from oral and rest of them were unique in BALF.

To explore the origins of the lung microbiome, we constructed a reference gene catalog based on the HOMD and HMP (https://portal.hmpdacc.org) databases. We compared annotated genes with the reference database to determine functional gene sources. According to our results, the majority of genes were shared, 32.84% genes were derived from oral, and 1.32% genes were derived from the gut, as determined by V search (see the Methods section for details) and the results were shown in Figure 4A. We also analyzed the crosstalk of the different enriched genes between smoking group and IPF group: 38% of them were from oral and rest of them were unique in BALF (Figure 4B). None of the different enriched genes were from gut.

### Functional Analysis of the Lung Microbiome

**Figure 5:**
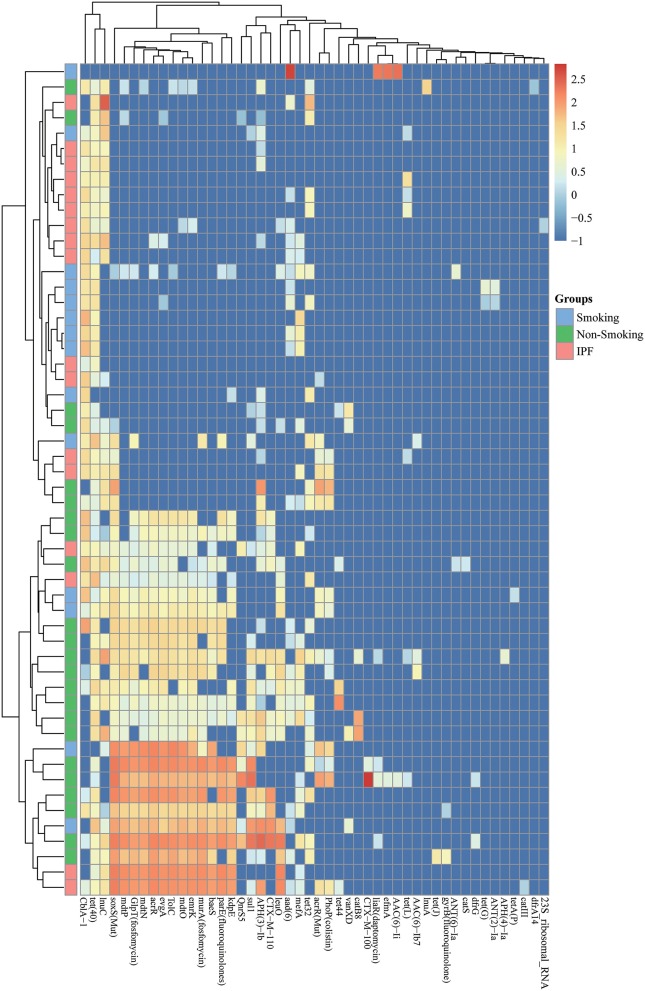
Abundances of antibiotic resistant genes from BALF samples. Each column corresponds to an individual BALF's sample from IPF group (pink color) and healthy control group (smoking: blue color; non-smoking: green color) showed on the left side. The types of antibiotic resistant genes were indicated in the boxes at the bottom. Each row corresponds to a specific abundance of antibiotic resistant genes based on different colors according to different abundance folds.

**Figure 6:**
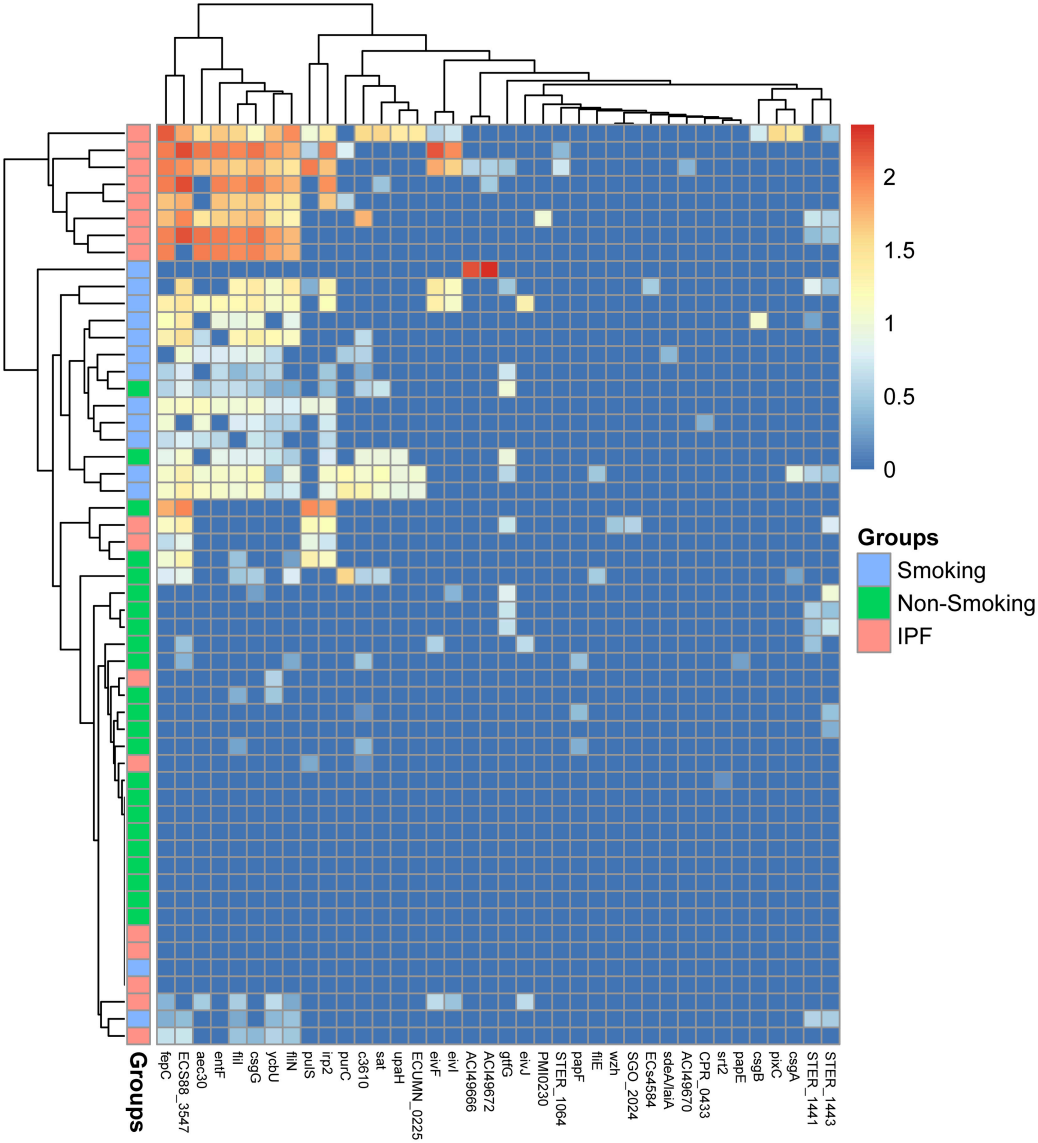
Abundances of virulence genes from BALF samples. Each column corresponds to an individual BALF's sample from IPF group (pink color) and healthy control group (smoking: blue color; non-smoking: green color) showed on the left side. The types of virulence factors were indicated in the boxes at the bottom. Each row corresponds to a specific abundance of virulence genes based on different colors according to different abundance folds.

To identify functional differences in the BALF microbiome between control subjects (including smokers and non-smokers) and IPF patients, shotgun metagenomics data were annotated using the KEGG and eggNOG database and analyzed for functional and metabolic pathways (Supplementary Tables 3, 4). A total of 2,707 KOs were identified either in healthy controls or IPF samples. We further mapped these KOs to KEGG modules and pathways and calculated the adjusted *P*-value for the hypergeometric distribution. In particular, ABC transporter systems, the two-component regulatory system, biofilm formation, methane metabolism, aromatic compound degradation, amino acid biosynthesis (including alanine, tyrosine, valine and leucine), vitamin biosynthesis, biotin metabolism, and amino sugar and nucleotide sugar metabolism were enriched in the IPF group (Supplementary Figure 2). Among these pathways, the ABC transporter system, which imports amino acids, represented the majority of the enriched genes in the IPF group (Supplementary Figure 3). Multidrug efflux genes, which contribute to drug resistance, were enriched in the IPF group. According to ARDB databases, antibiotic resistant genes (ARG) was checked and 48 different types of ARGs was identified, which showed in Supplementary Table 5. The relative abundance of identified ARGs in IPF group and healthy control groups (smoking and non-smoking) was shown in Figure 5. Compared with microbial genes from healthy control subjects, genes from IPF group were enriched of ARGS (5/10), including *tet*40, *acr*R, *evg*A, *tol*C, *mdt*O, with significantly abundance changes. These ARGs identified in IPF group are related to multidrug efflux, suggesting that the multidrug efflux pumps maybe the primary mechanism for bacteria to extrude antibiotics and other materials (Figure 5A). At the same time, virulence genes were also detected in this research showed in Supplementary Table 6 and the relative abundance of each group was showed in Figure 6.

## Discussion

The Human Microbiome Project was launched in 2007 to study the microbial inhabitants of the human body and the microbes that live on human mucosal surfaces with the understanding that human biology may partially depend on interactions with these microorganisms (Turnbaugh et al., 2007). Because the human lung was believed to be sterile, it was not included in the original Human Microbiome Project. However, a growing number of studies have revealed the presence of microbial communities in the lungs. Next generation sequencing technology has dramatically improved lung microbiome identification in healthy subjects and in patients with chronic airway diseases, but humanized genomic DNA contamination remains a major technical challenge (Corless et al., 2000; Zoetendal et al., 2006; Salonen et al., 2010; Wu et al., 2010). As shown in our previous study, the use of pure microbial DNA avoids humanized genomic pollution and facilitates investigation of the lung microbiota (Wen et al., 2016).

In our current study, bacteria and virus microbial genes discriminated between IPF patient BALF and the BALF of healthy individuals with a high specificity; for example, *Acinetobacter* and *Neisseria* were identified at the genus level. The most common pathogenic bacteria in the IPF group were *Acinetobacter* and *Neisseria* (at the genus level), and the most common pathogenic viruses were microvirus, simplex virus and varicella virus. The same results had been reported with an increased bacterial load and decreased microbial diversity in BAL samples from IPF patients (Morris et al., 2014). At the same time, there are clear differences in the IPF BAL microbiome (increase in *Neisseria, Streptococcus* and some other species) compared to healthy subjects, which was also proven in our study. The bacterial load affects survival in these patients whereas retrospective analysis of the lung microbiome in BAL samples from the COMET-cohort suggested that presence of specific *Streptococcus* operational taxonomic units (OTU) or *Staphylococcus* OTU was associated with worse outcomes of IPF. These studies were showed the differential microbiome and specific microbial genes changes in BALF isolated from IPF patients, which suggested microbiota-targeted biomarkers may be potential tools for disease diagnosis and predicting prognosis. Biodiversity analysis revealed greater microbiome diversity associated with better lung function in the control group; conversely, the IPF group demonstrated decreased microbial diversity in terms of bacterial gene richness. Analysis of BALF microbiome revealed the appearance of common oral and gut inhabitants in BALF. Notably, shifts in the bacterial make-up of the BALF were associated with the human gut and oral microbiotas. In the IPF group, the majority of inhabitants were derived from the oral microbiome, suggesting they shifted from the mouth and throat. Comparison of BALF microbial genes to oral and gut microbiomes demonstrated that majority genes were from oral source, which hint the potential mechanism of oral aspiration as a risk factor in pathogenesis of IPF.

This study provides an opportunity to identify the bacterial functions required for a bacterium to thrive in the context of the BALF from IPF group and healthy subjects. To identify the functions of the BALF genome, gene length and copy number were normalized, and the relative frequencies of different functions were deduced based on the number of genes recruited to different EggNOG clusters. ABC transporter systems, the two-component regulatory system, biofilm formation, amino acid and vitamin biosynthesis, and central carbon metabolism were enriched in the IPF group. Not surprisingly, projection of the range clusters in KEGG metabolic pathways provided highly explicit directions associated with antimicrobial resistance. Antimicrobial resistance is a natural response of bacteria to antibiotic exposure (Sherrard et al., 2014). Antimicrobial resistance may be intrinsic to a bacterium, arise from spontaneous genetic mutations, or be associated with horizontal gene transfer (Sherrard et al., 2014). The enrichment of ABC transporters and biofilm formation signals indicates the presence of major antimicrobial resistance pathways in the IPF lung.

ABC transporter systems play a large variety of biological roles in processes such as translation, elongation, and DNA repair. In some antibiotic-producing or drug-resistant bacteria, particularly Gram-negative bacteria, ABC systems are responsible for the active efflux of drugs and other harmful compounds across the cell envelope (Seeger and Van Veen, 2009). Relative abundance of multidrug efflux genes were increased in IPF group compared with healthy controls which listed in this research. Furthermore, the virulence factors also evaluated in this research and showed significant differences between IPF group and control group. Biofilm formation is a feature of chronic airway infection. Bacteria growing in biofilms are embedded in a matrix of exopolymeric substances and are much more resistant to antibiotics than organisms growing planktonically (Stewart and Costerton, 2001; Wu et al., 2015). Together, these findings emphasize antimicrobial resistance as a primary function of the BALF ecosystem.

Our descriptions of the major characteristics of the lung microbiome provide a holistic view to understand IPF patients. In our study, intrinsic changes in the microbiome emphasize the production of strong antimicrobial resistance by the BALF ecosystem, which is important to understand pathophysiological processes. The interesting modules and pathways found in this project suggest the BALF microbiome in IPF patients is related to antibiotic resistance. Therefore, more therapeutic options for IPF may become available through the adaptation of lung environments or the identification of beneficial probiotic microorganisms. Further studies in this area will lead to a deeper understanding of bacterial life in the BALF.

## Ethics Statement

Institutional Review Board and Ethics Committee of Beijing Hospital.

## Author Contributions

CW, YL, FX, and XT designed the study, interpreted the results, and drafted the manuscript. FS and SP analyzed the data. HD, TY, XX, QX, and KH made substantial contributions to the experimental protocol. HX, LZ, and WZ participated in sample preparation and DNA extraction. CW, YL, XT, and FS critically revised the manuscript for intellectual content. All authors participated in discussing and interpreting the results and gave final approval for manuscript submission.

### Conflict of Interest Statement

SP was employed by Annoroad Gene Technology (Beijing) Co., Ltd, Beijing, the People's Republic of China. The remaining authors declare that the research was conducted in the absence of any commercial or financial relationships that could be construed as a potential conflict of interest.

## Supplementary Material

Supplementary Figure 1 The workflow of this research was shown in this figure.

Supplementary Figure 2 Functional analysis of lung microbiome. The shotgun metagenomics data were annotated using the KEGG databases and analyzed at the functional and metabolic pathway levels. The KOs to KEGG modules and pathways were mapped to identify the BALF microbial functional differences between control subjects. The blue and red lines denote the enriched KEGG pathways in the control and IPF subjects, representatively.

Supplementary Figure 3 Enrichment of ABC transporter systems based on the knowledge of KEGG database. The red boxes stand for the enriched genes in BALF samples. The blue boxes stand for the rest genes from KEGG database.

Supplementary Table 1 Sequencing statistic of all BALF samples.

Supplementary Table 2 Taxonomy composition at different levels.

Supplementary Table 3 KEGG functional enrichment analysis.

Supplementary Table 4 EggNOG functional enrichment analysis.

Supplementary Table 5 Antibiotic resistant genes abundance comparison between different conditions.

Supplementary Table 6 Virulence genes abundance comparison between different conditions.
