# Supplementary figures and images for: Alterations to the Lung Microbiome in Idiopathic Pulmonary Fibrosis Patients
Source: Front Cell Infect Microbiol. 2019 May 21;9:149. doi: 10.3389/fcimb.2019.00149 (PMC6536613; doi:10.3389/fcimb.2019.00149)

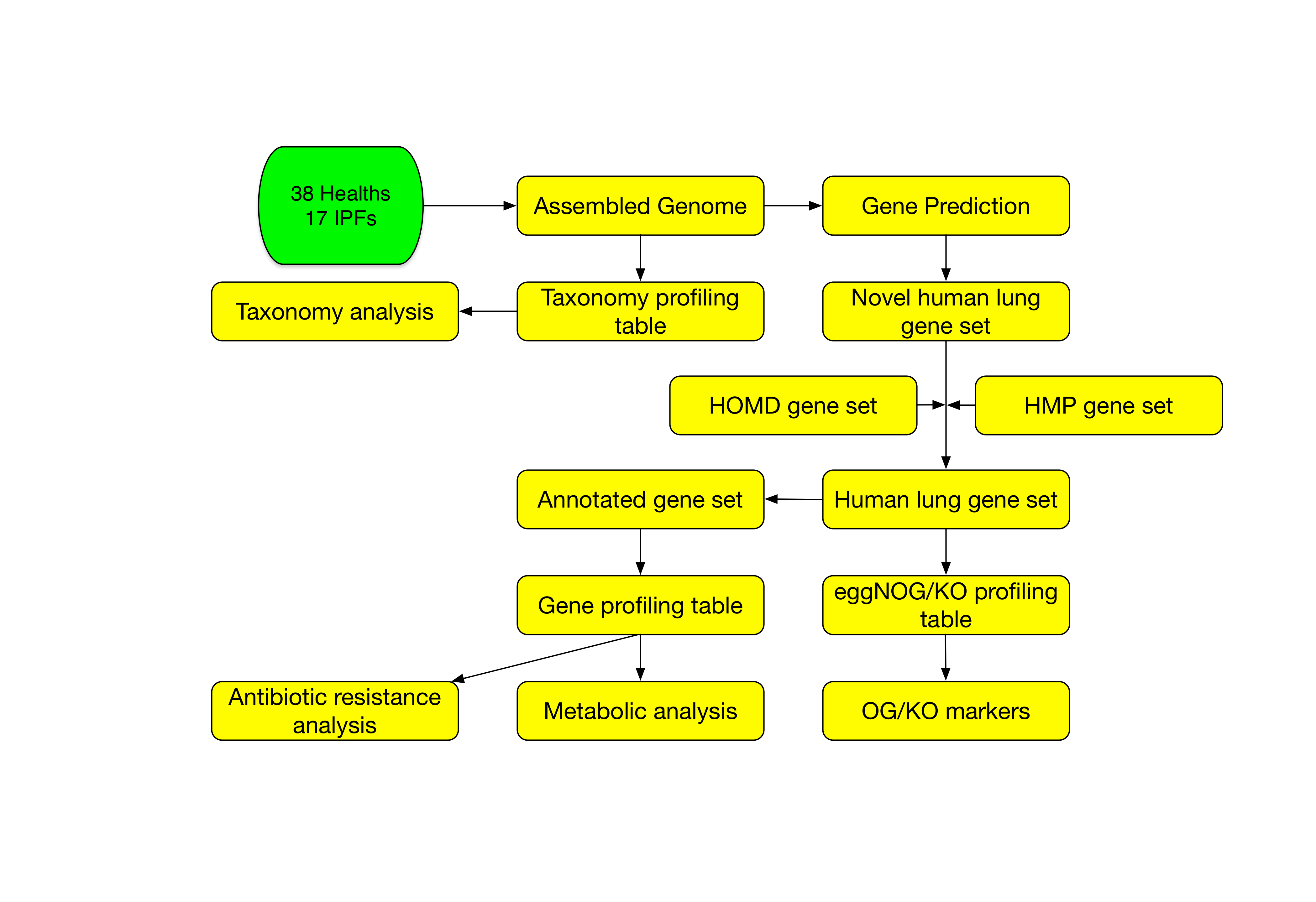

Supplement: Supplementary Figure 1 — The workflow of this research was shown in this figure. [file Image_1.PNG]

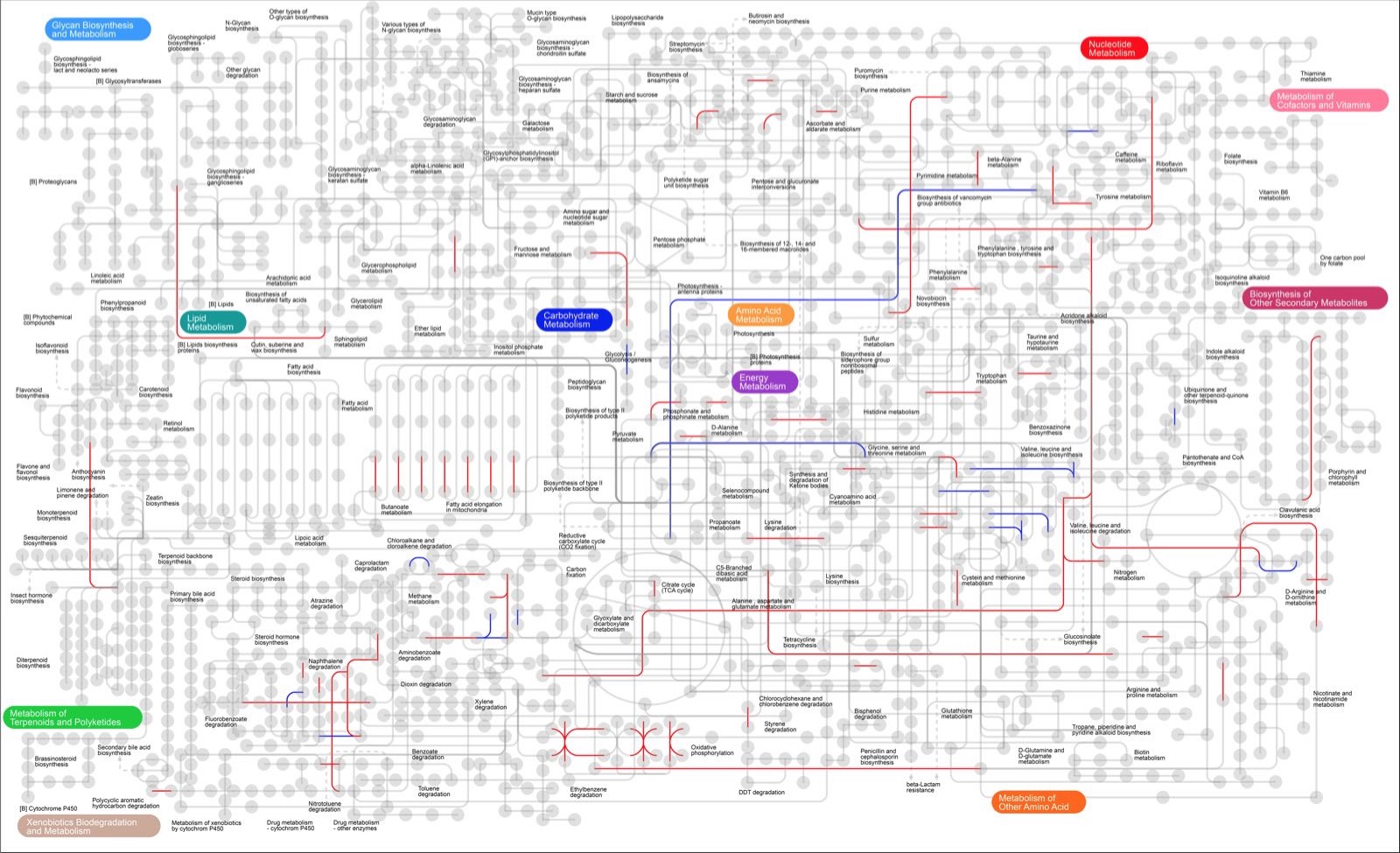

Supplement: Supplementary Figure 2 — Functional analysis of lung microbiome. The shotgun metagenomics data were annotated using the KEGG databases and analyzed at the functional and metabolic pathway levels. The KOs to KEGG modules and pathways were mapped to identify the BALF microbial functional differences between control subjects. The blue and red lines denote the enriched KEGG pathways in the control and IPF subjects, representatively. [file Image_2.JPEG]

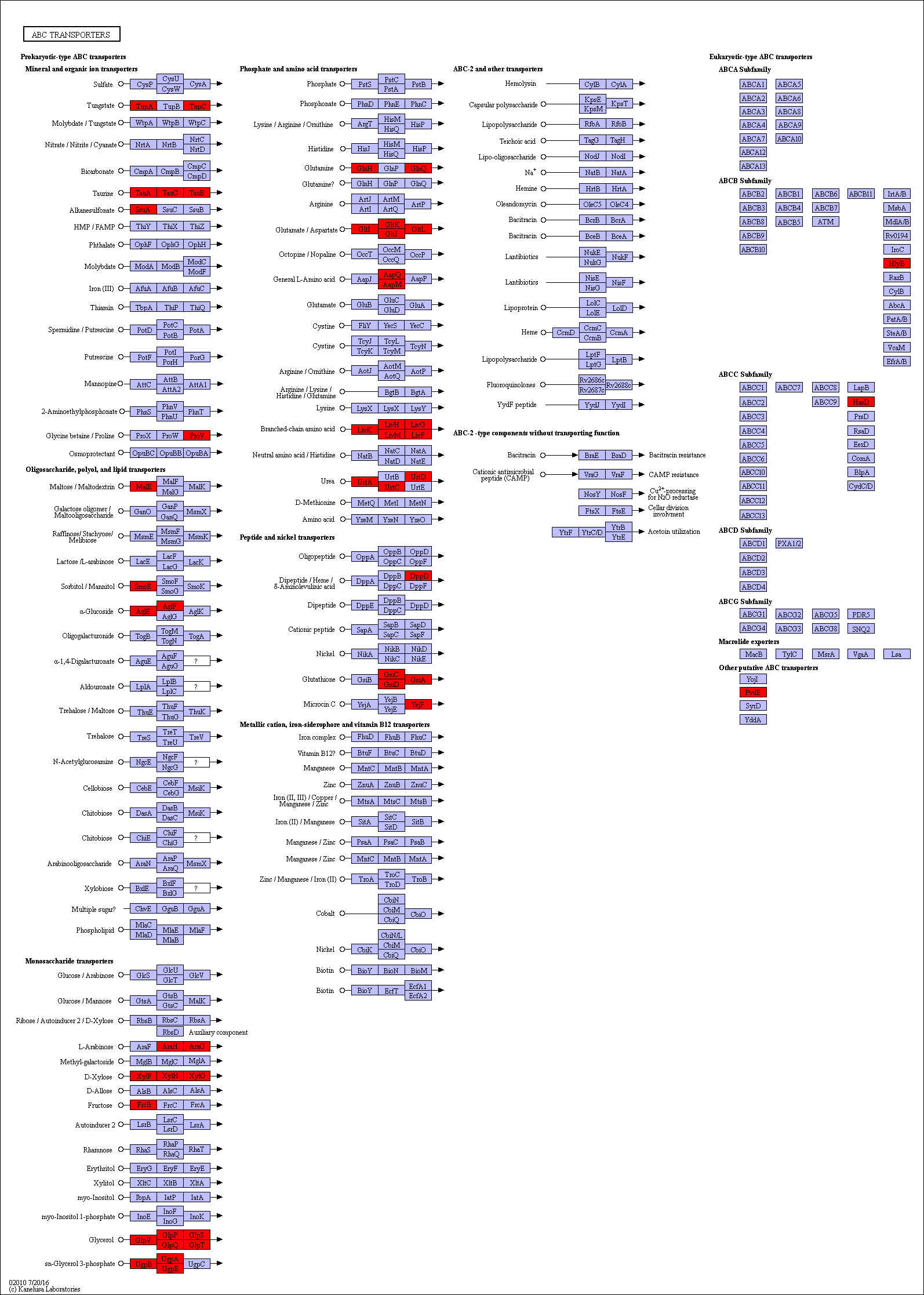

Supplement: Supplementary Figure 3 — Enrichment of ABC transporter systems based on the knowledge of KEGG database. The red boxes stand for the enriched genes in BALF samples. The blue boxes stand for the rest genes from KEGG database. [file Image_3.PNG]
